# Prevalence and Risk Factors Associated with Chronic Occupational Low Back Pain among Healthcare Professionals Working at Hospitals: Exploratory Survey Study

**DOI:** 10.1055/s-0044-1786729

**Published:** 2024-06-22

**Authors:** Otaviano de Oliveira, Breno Vasconcelos Brandão, David Bastos Vieira da Fonseca, Núbia Carelli Pereira Avelar, Janaine Cunha Polese, Amanda Aparecida Oliveira Leopoldino

**Affiliations:** 1 Departamento de Medicina, Faculdade de Ciências Médicas de Minas Gerais (FCMMG), Belo Horizonte, MG, Brasil; 2 Departamento do Futebol Profissional, Clube Atlético Mineiro, Belo Horizonte, MG, Brasil; 3 Curso de Medicina, Faculdade de Ciências Médicas de Minas Gerais (FCMMG), Belo Horizonte, MG, Brasil; 4 Departamento de Fisioterapia, Universidade Federal de Santa Catarina (UFSC), Araranguá, SC, Brasil; 5 Programa de Pós-Graduaéão em Ciências da Saúde, Faculdade Ciências Médicas de Minas Gerais, Belo Horizonte, MG, Brasil

**Keywords:** back pain, health professionals, low back pain, quality of life, work capacity evaluation

## Abstract

**Objective** This study aimed to describe the methodological process for developing a questionnaire to identify the prevalence and risk factors for chronic occupational low back pain in healthcare professionals working at hospitals.

**Method** An exploratory crossectional survey study was carried out in Belo Horizonte, MG, Brazil, and its metropolitan region, in two stages. Initially, the authors prepared a questionnaire based on the Roland Morris disability questionnaire and sent it to a committee of low back pain specialists for validation using the Delphi technique. The second stage consisted of sending the final questionnaire to health professionals working in a hospital environment for at least 2 years and presenting chronic low back pain for at least 3 months.

**Results** Validation occurred in two rounds of questionnaire adjustments by a panel consisting of physical therapists and physician experts in the field (orthopedists with more than 3 years of experience). Both rounds had 13 participants. The questionnaire initially consisted of 27 items, and, after validation, it had 19 items. The study included 65 subjects, with an average age of 40.91 years old and an average time working at a hospital of 40 hours per week. The total sample had 76.9% of physicians, 10.8% of physical therapists, and 12.3% of nurses or nursing technicians. Most (52.3%) subjects reported staying in uncomfortable positions affecting the lower back for 5 to 10 hours per day.

**Conclusion** We developed and validated, using the Delphi technique, a questionnaire on the prevalence and risk factors associated with chronic occupational low back pain among healthcare professionals working at hospitals. This unprecedented tool can benefit the population studied since the questionnaires currently used to evaluate chronic low back pain are not specific for investigating the occupational cause of this condition.

## Introduction

Low back pain is a common symptom and the major cause of disability in the world. 1 n its occupational variant, the condition appears or worsens due to the subject's work. Occupational low back pain must not be analyzed only as a medical issue but also as a socioeconomic problem as it affects the economically active population and is related to work incapacity. 2 Healthcare professionals working at hospitals need agility and face threats, risk of infections, and increasing demands on medical skills. In this sense, this category must suffer from low back pain resulting from work, and this effect remains unknown.

Simsek et al. 3 demonstrated that the lifetime prevalence of low back pain among healthcare workers was 53%, with an annual one of 39%, and a specific one of 29.5%. Furthermore, the literature identified three large groups of potential risk factors for low back pain: (a) individual factors such as body weight and age; (b) mechanical factors such as heavy physical load, lifting, crooked postures, and vibration; and (c) psychosocial factors such as control and job satisfaction. 4 These psychosocial factors affect physical and mental performance at work but also influence medical errors. 1

Few epidemiological studies have analyzed the onset and risk factors for low back pain among healthcare professionals. We found a single study about it, showing its prevalence among nurses in Africa as 70%. 4 The assessment tools for low back pain present high heterogeneity in international epidemiological studies. A systematic review of 165 studies from 54 countries found that the average one-month prevalence of low back pain was 30.8%, with a standard deviation of 12.5%. The deviation for the one-year prevalence was even higher. 5

Questionnaire use in the medical field is widespread. Although several questionnaires have been developed to assess disability and activity limitations in patients with low back pain, 6 the literature has no specific method for assessing the occupational cause of this condition in healthcare professionals.

For this reason, the present study aimed to develop and describe the methodological process for producing a questionnaire using the Delphi technique, with the help of low back pain specialists, targeted at identifying the prevalence and risk factors for chronic occupational low back pain healthcare professionals working in hospitals.

## Materials and Methods

### Study Design, Sample, and Ethical Aspects

This study is an exploratory crossectional survey. An expert committee validated the items in the developed questionnaire. The inclusion criteria for these professionals were physical therapists or orthopedists with expertise in low back pain. The inclusion criteria for questionnaire respondents in the second stage of the study were healthcare professionals working in a hospital environment for at least 2 years and presenting chronic low back pain for at least 3 months.

The developed questionnaire investigated contextual factors associated with occupational low back pain in clinical and surgical practice at the tertiary level. The final questionnaire was sent by email to healthcare professionals from the city of Belo Horizonte between February and July 2022. These professionals were recruited from the general population using a prior research list.

The Research Ethics Committee evaluated and approved the protocol for this study under opinion number 5.003.882. All participants signed an informed consent form.

### Development and Application of the Questionnaire

A previous questionnaire on low back pain, the Roland Morris Disability Questionnaire, was the basis of our questionnaire since it evaluates and quantifies low back pain as a score to increase diagnostic precision and guide the necessary treatment. This tool assesses the physical limitations resulting from reported lumbar spine pain and consists of 24 “yes” or “no” questions regarding symptoms on the evaluated day, describing the back pain situation. Nusbaum et al. 7 translated, adapted, and validated it for the Brazilian population, allowing its usual application in interviews.

It is an easy and quick questionnaire, taking an average of five minutes to administer, a significant factor in choosing it as a reference. This questionnaire evaluates different clinical parameters to assess the disabilities caused by low back pain and its consequences on quality of life. Scores range from 0 to 24 per the sum of positive responses, with higher scores indicating higher disability. 7 It presents high internal consistency (Cronbach α = 0.92) and interrater reliability with an intraclass correlation coefficient (ICC) of 0.95 and a 95% confidence interval (95% CI = 0.93–0.97), showing a good correlation with other pain scales. 8

Therefore, after evaluating this questionnaire's positive and negative points, we created the first version of our questionnaire on the prevalence and risk factors associated with chronic occupational low back pain among healthcare professionals working at hospitals. This version carefully evaluated a specific type of low back pain in a well-defined population. To this end, we included pertinent questions to better diagnose the type of low back pain and its prevalence in the mentioned population, considering its peculiarities.

We used the Delphi technique to validate the final version of our questionnaire. This technique consists of an interactive estimation method to establish the content validity of a tool by systematically analyzing the opinions of experts on a given subject. After rounds of theoretical content analysis, in which experts shared their answers, the group reached a consensus. 9

### Data Analysis

We estimated the prevalence of responses to the proposed questionnaire using percentages. The descriptive analysis of the study sample employed measures of central tendency (mean and standard deviation) for continuous variables and absolute (n) and relative frequency (%) for categorical variables. We performed all analyses using the Statistical Package Social Sciences (SPSS, IBM Corp., Armonk, NY, USA) software, version 26.0.

## Results

### Questionnaire Validation using the Delphi Technique

**Fig. 1:**
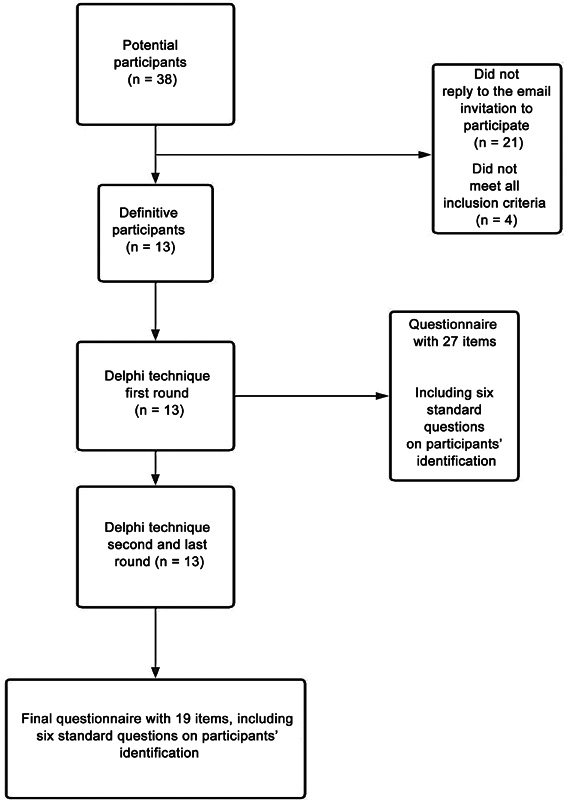
Flowchart of the Delphi technique's application for questionnaire validation.

For the first round of the Delphi technique, we sent the questionnaire electronically; for the second round, we sent it electronically and in person. The themes selected in the review guided the initial questionnaire preparation. We launched the questionnaire initially via Google Forms to 38 potential participants, as illustrated in Fig. 1 . Of these, 4 declared themselves ineligible to answer the questionnaire because they did not meet all the inclusion criteria, and 21 did not answer the request for participation in the Delphi technique. In the first round, the panel of experts consisted of 13 participants, and the questionnaire had 27 items, with the initial 6 referring to the identification of the participants.

In the second and last round, the panel had the same 13 participants. These experts suggested excluding eight questions between the first and second rounds of the Delphi technique. Of these, 6 were not consensual regarding the importance of the final score/diagnosis, and 2 did not reach a consensus for requiring a specific orthopedic terminology, a potential factor for reducing reproducibility on a large scale.

Thus, the final questionnaire had 19 items, again with the initial six items referring to identification, with suggestions for changing the vocabulary in six items (number 5 in the Identification section and numbers 2, 3, 4, 6, and 8 in the Validation section), with consensus on all items after changes. The items from this round comprise the final version of the questionnaire.

### Application of the Final Version of the Questionnaire

The validated questionnaire ( Box 1 ) was applied electronically via the Google Forms platform to 65 healthcare professionals (physicians, physical therapists, nurses, and nursing technicians). Our sample consisted entirely of healthcare professionals working in hospitals for at least 2 years and presenting chronic low back pain with symptoms for at least 3 months.

**Chart 1:** Questionnaire on occupational chronic low back pain among healthcare professionals working at hospitals

**In a typical week, how many hours do you work in a hospital setting?**	40 hours or more 43.1%	30–40 hours 23.1%	20–30 hours 18.5%	20 hours 15.4%	
**Do you stay in the same position (e.g., sitting down or standing up) during most of your working day in a hospital environment?**	Yes 80%	No 20%			
**During a shift (at least 12 hours) and/or typical workday, how long do you remain in uncomfortable positions affecting your lower back?**	0–4 hours 36.9%	5–10 hours 52.3%	10–20 hours 10.8%		
**Do you change position frequently during a procedure to have more comfort in your lower back?**	Yes 72.3%	No 27.7%			
**Because of your pain in the lower back, do you avoid standing up or walking?**	Yes 13.8%	No 53.8%	Sometimes 32.3%		
**Because of your lower back pain, do you avoid using stairs or ramps?**	Yes 12.3%	No, 78.5%	Sometimes 9.2%		
**Does your lower back pain radiate to your lower limbs?**	Yes 6.2%	No, 76.9%	Sometimes 16.9%		
**Does your lower back pain show signs of paresthesia (tingling)?**	Yes 6.2%	No, 86.2%	Sometimes 7.7%		
**How intense is your low back pain while you fill out the questionnaire from 0 to 10? (With 0 being none and 10 being the worst pain you have ever felt)**	Sample average: 2.63 on the pain scale used				
**What is the average intensity of low back pain in the last 6 weeks from 0 to 10? (With 0 being none and 10 being the worst pain you have ever felt)**	Sample average: 4.07 on the pain scale used				
**Have you been taking any medication for lower back pain over the last 3 months? How often do you take it?**	Yes, less than once a week 18.5%	Yes, once or twice a week 6.1%	Yes, 3–5 times a week 1.5%	Yes, daily 4.6%	No 69.2%
**Have you ever visited a specialist due to low back pain?**	Yes 49.2%	No 50.8%			
**Do you perform any supplementary interventions due to low back pain? (e.g., physical therapy, acupuncture, physical exercises at a gym, yoga etc.)**	Yes 58.5%	No 41.5%			

### Participant Identification

Of the 65 research participants, 40 (61.5%) were men and 25 (38.5%) were women. As for profession, 50 (76.9%) participants were physicians, 7 (10.8%) were physical therapists, 4 (6.2%) were nurses, 3 (4.6%) were nursing technicians, and 1 (1.5%) was from another healthcare area. The maximum degree selected was Specialization and/or Residency, representing 38 (58.5%), Professional or Academic Master's degree 14 (21.5%), Doctorate 6 (9.2%), Bachelor's degree 4 (6.2%), Technician 2 (3.1%), and Academic Professor/Researcher 1 (1.5%). Concerning the weekly workload in a hospital environment, 28 (43.1%) worked 40 hours or more, 15 (23.1%) 30 to 40 hours, 12 (18.5%) 20 to 30 hours, and 10 (15.4%) worked up to 20 hours ( Table 1 ).

**Table 1:** Characterization of the sample responding to the questionnaire developed in the study (n = 64)

**Average age (years)**	40.91
**Male/female gender, n (%)**	40/25 (61.5/ 38.5)
**Professional qualification**	
Medicine, n (%)	50 (76.9)
Physical therapy, n (%)	7 (10.8)
Nursing or nursing technician, n (%)	7 (12.3)
**Highest Degree**	
Bachelor's degree or Specialization, n (%)	45 (69.3)
Master's or Doctorate, n (%)	20 (30.7)

### Questionnaire Responses

Of the study's 65 participants, 52 (80%) subjects reported remaining in the same position (sitting down or standing up) for most of their working hours in a hospital environment. During a shift (minimum of 12 hours) and/or a typical working day, 7 (10.8%) participants remained in uncomfortable positions affecting the lower back for 10 to 20 hours, 34 (52.3%) from 5 to 10 hours, and 24 (36.9%) from 0 to 4 hours. Furthermore, 47 (72.3%) subjects reported the need to change positions frequently during a procedure to have more comfort in the lower back.

We asked the participants if they avoided standing up or walking because of low back pain; 9 answered “yes”, 35 (53.8%) said “no”, and 21 (32.3%) responded “sometimes”. Also, 8 (12.3%) avoided using stairs or ramps due to the pain, while 51 (78.5%) denied avoiding it and six (9.2%) answered “sometimes”.

When asked about irradiation to the lower limbs, 4 (6.2%) responded “yes”, 11 (16.9%) said “sometimes”, and 50 (76.9%) answered “no”.

Regarding signs of paresthesia in low back pain, 4 (6.2%) answered “yes”, 56 (86.2%) stated “no”, and 5 (7.7%) said “sometimes”. The intensity of low back pain, from 0 to 10, at the time of completing the questionnaire was 0 (0.0%), 1 (47.7%), 2 (9.2%), 3 (15.4%), 4 (7.7%), 5 (7.7%), 6 (6.2%), 7 (4.6%), 8 (1.5%), 9 (0.0%), and 10 (0.0%). The average intensity of low back pain in the last 6 weeks, from 0 to 10, was 0 (0.0%), 1 (15.4%), 2 (15.4%), 3 (15.4%), 4 (15.4%), 5 (10.8%), 6 (9.2%), 7 (7.7%), 8 (7.7%), 9 (1.5%), and 10 (1.5%).

About the frequency of use of medication for low back pain in the last 3 months, 45 (69.2%) participants responded that they do not use it, 12 (18.5%) used it less than once a week, four (6.2%) used it once or twice a week, one (1.5%) used 3 to 5 times a week, and three (4.6%) used it daily. Furthermore, 32 (49.2%) participants had already consulted a specialist due to low back pain, and 38 (58.5%) performed some supplementary intervention (physical therapy, yoga, acupuncture, gym) due to low back pain ( Chart 1 ).

## Discussion

The present study provided an efficient method of evaluating the prevalence and risk factors of occupational low back pain in healthcare professionals at the hospital level. A literature review reported the methodological quality of the Rolland Morris questionnaire was good. Thus, due to its high dissemination in the literature, easy applicability, and low cost, it was used as a guide for developing the occupational low back pain assessment questionnaire for healthcare professionals working in a hospital environment, which was employed to evaluate the degree of involvement of low back pain of occupational origin in this group. 9 10

Occupational low back pain is the leading cause of disability in the world and one of the most prevalent complaints in primary care in Brazil. It is the most frequently reported spinal complication and is often inadequately or inefficiently managed. Healthcare professionals are usually exposed to risk factors for this condition due to excessive workload and inadequate posture. 8 Current research on occupational low back pain focuses on biomechanical factors and psychosocial variables that gained importance over time. 11

Low back pain is a physical and psychosocial pathology. As a result, prospective cohort studies indicate that low work satisfaction and appreciation and excess stress at work from high demands and long working hours showed a significantly increased odds ratio (OR) for the prevalence of low back pain. 12 Thus, specialists guide their treatment indications based on radiographic findings (presence of osteoarthritis, neuropathy, or both) and the pain scale, represented in the Roland Morris disability questionnaire. However, we believe this classification often does not consider the peculiarities of occupational low back pain, along with having weak or moderate reproducibility criteria, covering a portion of patients who are underdiagnosed, inappropriately treated, or both. 13 14

Diagnosis for this condition is simple, relying on a characteristic clinical picture and imaging tests. However, the high-demand routine of healthcare professionals and inadequate body posture make occupational back pain management difficult since causal factors are unlikely to change. Thus, this condition presents a constant challenge for healthcare professionals at the tertiary level. 15

The key issue in managing any disease is defining the best indication for each type of treatment. Questionnaires in clinical practice aim to stratify each case according to the stage of disease evolution, therefore allowing the determination of the best treatment. Therefore, a specific questionnaire for occupational low back pain among healthcare professionals at the hospital level is a simple and inexpensive way to classify the severity of the condition and assess the presence of a direct relationship between the harmful factors inherent to long-term work at a tertiary level, remaining in an orthostatic position for prolonged hours, poor adherence to physical exercise, and disease worsening. This information, along with the knowledge of the sociodemographic profile of the target group, assure better preventive and/or specialized therapeutic intervention. 16

Disease-specific questionnaires are often considered superior to their generic counterparts for clinical applications. In turn, these are more appropriate when comparing different diseases or evaluating types of care across disease categories. 17 Therefore, although there are extensive classifications and studies on the prevalence of low back pain in Brazil, we realized there is no exclusive standardization for occupational low back pain in healthcare professionals at the hospital level.

In previous studies, work-related musculoskeletal pain was highly prevalent among healthcare professionals working in hospitals. 18 19 20 These pain conditions often relate to physical effort and psychological stress. However, a study with health professionals from a hospital in Switzerland showed poor posture at work as the main factor resulting in pain. 19 Likewise, we observed that maintaining the same posture for long periods and the discomfort from these positions are quite common in our sample.

Despite the notable exposure to risk factors for musculoskeletal pain, studies show a wide variety among different types of healthcare professionals. Furthermore, there is variability from study to study according to the questions asked and the metric used. 20

This study has some limitations. The sample was limited to healthcare professionals working in the metropolitan region of Belo Horizonte, and it may not reflect the behavior of professionals from other places. Furthermore, the validated questionnaire was administered to 65 participants. However, the primary objective of the present study was to develop an expert-validated questionnaire.

## Conclusion

The two-step Delphi technique contributed to developing and validating a questionnaire on the prevalence and risk factors associated with chronic occupational low back pain among healthcare professionals working at hospitals. The tool proved to be valid and easy to apply. Furthermore, after applying the final version of the questionnaire, we determined the prevalence and risk factors associated with low back pain, which can be used for both clinical or research purposes.
