# Supplementary material for: Prevalence and Risk Factors Associated with Chronic Occupational Low Back Pain among Healthcare Professionals Working at Hospitals: Exploratory Survey Study
Source: Rev Bras Ortop (Sao Paulo). 2024 Jun 22;59(3):e378–84. doi: 10.1055/s-0044-1786729 (PMC11193593; doi:10.1055/s-0044-1786729)
Supplement: Related texts

# Research article: Prevalência e fatores de risco associados a dor lombar ocupacional crônica entre profissionais da área da saúde que atuam em nível hospitalar: Estudo exploratório

**Keywords:** avaliação da capacidade de trabalho, dor lombar, dor nas costas, profissionais de saúde, qualidade de vida

## Abstract

**Objetivo** Este estudo pretende descrever o processo metodológico para a elaboração de um questionário para identificar a prevalência e os fatores de risco associados à dor lombar ocupacional crônica nos profissionais da área da saúde que atuam em nível hospitalar.

**Método** Foi realizado um estudo transversal exploratório do tipo questionário. O estudo foi realizado na cidade de Belo Horizonte e região metropolitana, em duas etapas. Inicialmente foi elaborado pelos autores um questionário baseado no questionário de deficiências Roland Morris e enviado a um comitê de especialistas em lombalgia para validação do mesmo através da técnica Delphi. A segunda etapa consistiu em enviar o questionário final a profissionais de saúde que atuam em ambiente hospitalar há pelo menos 2 anos e que tenham lombalgia crônica há pelo menos 3 meses.

**Resultados** A validação foi realizada em duas rodadas de adequações do questionário, com painel composto por fisioterapeutas e médicos especialistas na área (ortopedistas com mais de 3 anos de atuação). Ambas as rodadas contaram com 13 participantes. O questionário foi composto inicialmente por 27 itens e, após validação, 19 itens. O estudo incluiu 65 indivíduos, com idade média de 40,91 anos e tempo médio de atuação em nível hospitalar semanal de 40 horas. A amostra total possuía 76,9% médicos, 10,8% fisioterapeutas e 12,3% enfermeiros ou técnicos de enfermagem. A maioria (52,3%) dos indivíduos relatou manter-se em posições desconfortáveis que afetam a região lombar por 5 a 10 horas por dia.

**Conclusão** Foi desenvolvido e validado, pela técnica Delphi, um questionário sobre a prevalência e fatores de risco associados a dor lombar ocupacional crônica entre profissionais da área da saúde que atuam em nível hospitalar. Este instrumento inédito pode trazer benefícios para a população estudada, visto que os questionários utilizados atualmente para a avaliação de dor lombar crônica não são específicos para a investigação da causa ocupacional de tal condição.

## Introdução

Dor lombar é um sintoma bastante comum, sendo considerada a principal causa de incapacidade no mundo. 1 Sua variante ocupacional é aquela em que o surgimento ou a piora estão relacionadas ao ofício do indivíduo. A dor lombar ocupacional não deve ser analisada somente como uma questão médica, mas também como uma questão socioeconômica por acometer a população economicamente ativa e estar relacionada a quadros de incapacidade laborativa. 2 Profissionais da área da saúde que atuam a nível hospitalar estão expostos a necessidade de agilidade no trabalho, ameaças, risco de infecções e demandas crescentes de competências médicas. Nesse sentido, tal categoria deve sofrer de dor lombar decorrente do trabalho, e esse efeito é ainda desconhecido.

O estudo de Simsek et al. 3 demonstrou que a prevalência de dor lombar entre trabalhadores da área da saúde ao longo da vida era de 53%, a anual era de 39% e a pontual de 29.5%. Ademais, três grandes grupos de potenciais fatores de risco para dor lombar foram identificados na literatura: (a) fatores individuais como peso corporal e idade, (b) fatores mecânicos como carga física pesada, levantamento, posturas tortas, vibração e (c) fatores psicossociais como controle e satisfação do emprego. 4 Tais fatores psicossociais podem não só afetar a performance física e mental no trabalho, mas também na ocorrência de erros médicos. 1

Poucos estudos epidemiológicos analisaram o aparecimento e fatores de risco associados à dor lombar entre profissionais da saúde. Foi encontrada apenas uma pesquisa que estudou a prevalência entre enfermeiras na África e reportou uma prevalência de 70%. 4 Entre os instrumentos de avaliação para dor lombar, constata-se uma grande heterogeneidade nos estudos epidemiológicos internacionalmente. Uma revisão sistemática de 165 estudos de 54 países apontou que a prevalência média de um mês de dor lombar foi 30.8%, com desvio padrão de 12,5%. O desvio para a prevalência de um ano foi ainda maior. 5

Sabe-se que questionários são amplamente usados na profissão médica. Apesar de vários questionários terem sido desenvolvidos para avaliação de incapacidade e limitações de atividade em pacientes com dor lombar, 6 inexiste na literatura um método específico para avaliação da causa ocupacional dessa condição em profissionais da saúde.

Por este motivo, o presente estudo objetivou desenvolver e descrever o processo metodológico para a elaboração de um questionário por meio da técnica Delphi, com ajuda de especialistas em dor lombar, direcionado a identificar a prevalência e os fatores de risco associados à dor lombar ocupacional crônica em profissionais de saúde que atuam em ambiente hospitalar.

## Materiais e Métodos

## Delineamento do Estudo, Amostra e Aspectos Éticos

Foi realizado um estudo transversal exploratório, do tipo questionário *.* As questões do questionário desenvolvido foram validadas por um comitê de especialistas e os critérios de inclusão foram: ser fisioterapeuta ou ortopedista com expertise na área de lombalgia. Já os critérios de inclusão para os respondentes do questionário na segunda etapa do estudo foram: profissionais de saúde que atuem em ambiente hospitalar há pelo menos 2 anos e que tenham lombalgia crônica há pelo menos 3 meses.

O questionário desenvolvido investiga os fatores contextuais associados à dor lombar ocupacional na prática clínica e cirúrgica em nível terciário. O questionário final foi enviado via e-mail aos profissionais de saúde da cidade de Belo Horizonte entre fevereiro e julho de 2022. Os profissionais foram recrutados na sociedade em geral, por meio de lista de pesquisa prévia.

O protocolo deste estudo foi avaliado e aprovado pelo Comitê de Ética em Pesquisa, conforme parecer consubstanciado no número 5.003.882. Todos os participantes assinaram termo de consentimento livre e esclarecido.

## Desenvolvimento e Aplicação do questionário

As questões foram baseadas em um questionário prévio sobre dor lombar, o questionário de deficiência Roland Morris, devido a sua finalidade de avaliar e quantificar em uma escala o nível de dor lombar para auxiliar em um diagnóstico mais preciso e guiar o tratamento necessário. Este instrumento avalia as limitações físicas resultantes das dores referidas na coluna lombar e é composto por 24 questões de “sim” ou “não” a respeito de sintomas apresentados no dia avaliado, que descrevem a própria situação de dor nas costas. Foi traduzido, adaptado e validado para a população brasileira por Nusbaum et al. 7 e geralmente é aplicado por meio de entrevistas.

É um questionário de fácil e rápida aplicação, levando em média cinco minutos para ser administrado, fator importante na escolha deste como referência. Foi proposto para a avaliação de diferentes parâmetros clínicos no intuito de avaliar as incapacidades causadas pela dor lombar e suas consequências na qualidade de vida. Os escores variam de 0 a 24, que é dado pela soma das respostas positivas, sendo que escores maiores indicam maior incapacidade. 7 O questionário apresenta alta consistência interna (Cronbach α= 0.92) e confiabilidade interexaminadores com um coeficiente de correlação intraclasse (ICC) = 0.95, e um intervalo de confiança de 95% (IC 95% = 0.93–0.97), demonstrado uma boa correlação com outras escalas de dor. 8

Dessa forma, após avaliar os pontos positivos e negativos deste questionário, foi criado a primeira versão do questionário sobre a prevalência e fatores de risco associados a dor lombar ocupacional crônica entre profissionais da área da saúde que atuam em nível hospitalar. Este, com finalidade de avaliar criteriosamente um tipo específico de dor lombar e em uma população bem definida. Para tal, foram utilizadas questões pertinentes para melhor diagnosticar o tipo de dor lombar e sua prevalência na população citada, levando em conta suas peculiaridades.

A versão final foi validada por meio da técnica Delphi. Essa técnica consiste em um método interativo de estimativa, capaz de estabelecer a validade de conteúdo de instrumentos, ao analisar de forma sistemática as opiniões de especialistas em um determinado assunto. Foram realizadas rodadas de análises do conteúdo teórico, nas quais os especialistas compartilham suas respostas até que o grupo entre em um consenso. 9

## Análise dos Dados

As prevalências das respostas do questionário proposto foram estimadas usando porcentagens. A análise descritiva da amostra respondente do estudo foi realizada por meio de medidas de tendência central (média e desvio padrão) para as variáveis contínuas e por meio de medidas de frequência absoluta (n) e relativa (%) para as variáveis categóricas. Todas as análises foram realizadas utilizando o programa *Statistical Package Social Sciences* (SPSS, IBM Corp., Armonk, NY, USA) versão 26.0.

## Resultados

## Validação do Questionário pela Técnica Delphi

Durante a primeira rodada da técnica Delphi o questionário foi enviado eletronicamente; na segunda rodada, foi enviado eletrônica e presencialmente. Os temas selecionados na revisão nortearam a elaboração do questionário inicial. O questionário foi inicialmente lançado via Google Forms (Google LLC., Mountain View, CA, EUA) para 38 participantes, conforme ilustrado na Fig. 1 . Destes, 4 se declararam não elegíveis a responder o questionário por não serem aprovados em todos os critérios de inclusão e 21 por não apresentaram um retorno mediante envio da solicitação de participação da técnica Delphi. Na primeira rodada, o painel de especialistas foi composto por um total de 13 participantes, com questionário de 27 itens, com os 6 iniciais referentes à identificação dos participantes.

**Fig. 1:** Fluxograma da aplicação da técnica Delphi para validação do questionário.

Na segunda e última rodada, o painel foi composto pelos mesmos 13 participantes. Foi sugerido pelos especialistas a exclusão de 8 questões entre o primeiro e segundo *round* da técnica Delphi. Dessas, 6 não obtiveram consenso a respeito da importância para a pontuação/diagnóstico final e 2 não obtiveram consenso por necessidade de terminologia ortopédica específica, fator potencial para redução da reprodutibilidade em larga escala.

Assim, o questionário finalizou com 19 itens, novamente com os 6 itens iniciais referentes à identificação, havendo sugestão para alteração de vocabulário em 6 itens (número 5 da seção Identificação e números 2, 3, 4, 6 e 8 da seção Validação), com consenso em todos os itens após alterações. Os itens desta rodada seguiram para a escrita e desenvolvimento da versão final do questionário.

## Aplicação da Versão Final do Questionário

O questionário validado ( Quadro 1 ) foi aplicado eletronicamente por meio da plataforma Google Forms a 65 profissionais de saúde (médicos, fisioterapeutas, enfermeiros e técnicos de enfermagem). A amostra do presente estudo foi constituída inteiramente por profissionais de saúde que trabalham em nível hospitalar há pelo menos 2 anos e apresentam dor lombar crônica com sintomas por pelo menos 3 meses.

**Quadro 1:** Questionário sobre dor lombar ocupacional crônica entre profissionais da área da saúde que atuam em nível hospitalar

**Em uma semana típica, quantas horas você trabalha em ambiente hospitalar?**	40 horas ou + 43,1%	30–40 horas 23,1%	20–30 horas 18,5%	20 horas 15,4%	
**Você permanece em uma mesma posição (ex: sentada ou de pé) a maior parte do tempo de sua jornada de trabalho em ambiente hospitalar?**	Sim 80%	Não 20%			
**Em um plantão (no mínimo 12h) e/ou jornada típica de trabalho, quanto tempo você permanece em posições desconfortáveis que afetam a região lombar?**	0–4 horas 36,9%	5–10 horas 52,3%	10–20 horas 10,8%		
**Você muda de posição frequentemente durante a realização de um procedimento para ter mais conforto na região lombar?**	Sim 72,3%	Não 27,7%			
**Por causa da sua dor na região lombar, você evita manter-se em ortostatismo (posição de pé, ereta) ou deambular (caminhar)?**	Sim 13,8%	Não 53,8%	Às vezes 32,3%		
**Por causa da sua dor na região lombar, você evita o uso de escadas ou rampas?**	Sim 12,3%	Não 78,5%	Às vezes 9,2%		
**A sua dor lombar irradia para os membros inferiores?**	Sim 6,2%	Não 76,9%	Às vezes 16,9%		
**A sua dor lombar apresenta sinais de parestesia (formigamento)?**	Sim 6,2%	Não 86,2%	Às vezes 7,7%		
**Qual a intensidade de dor lombar no momento que está preenchendo o questionário de 0 a 10? (Onde 0 é nenhuma e 10 a pior dor que você já sentiu)?**	Média da amostra: 2,63 na escala de dor utilizada				
**Qual a média de intensidade de dor lombar nas últimas 6 semanas de 0 a 10? (Onde 0 é nenhuma e 10 a pior dor que você já sentiu)**	Média da amostra: 4,07 na escala de dor utilizada				
**Você está fazendo uso recorrente de algum medicamento para dor lombar nos últimos 3 meses? Com qual frequência você toma este medicamento?**	Sim, menos de 1 vez por semana 18,5%	Sim, de 1 a 2 vezes por semana 6,1%	Sim, de 3 a 5 vezes por semana 1,5%	Sim, diariamente 4,6%	Não 69,2%
**Já fez alguma consulta com especialista devido a dor lombar?**	Sim 49,2%	Não 50,8%			
**Você realiza alguma intervenção complementar devido a dor lombar? (Ex: fisioterapia, acupuntura, academia, yoga etc.)**	Sim 58,5%	Não 41,5%			

## Identificação dos Participantes

Dos 65 participantes da pesquisa, 40 (61,5%) eram homens e 25 (38,5%) mulheres. Quanto a profissão, 50 (76,9%) eram da área de Medicina, 7 (10,8%) de Fisioterapia, 4 (6,2%) de Enfermagem, 4 (4,6%) de técnica de enfermagem e 1 (1,5%) de outra área da saúde. A titulação máxima marcada foi Especialização e/ou Residência, representando 38 (58,5%), Mestrado Profissional ou Acadêmico 14 (21,5%), Doutorado 6 (9,2%), Bacharelado 4 (6,2%), Técnico 2 (3,1%) e Professor/Pesquisador Universitário 1 (1,5%). Em relação à carga horária de trabalho semanal em ambiente hospitalar, 28 (43,1%) exercem 40 horas ou mais, 15 (23,1%) de 30 a 40 horas, 12 (18,5%) de 20 a 30 horas e 10 (15,4%) exercem até 20 horas ( Tabela 1 ).

**Tabela 1:** Caracterização da amostra respondente do questionário desenvolvido no estudo (n = 64)

**Idade média (anos)**	40.91
**Sexo masculino/feminino, n (%)**	40/25 (61.5/ 38.5)
**Formação profissional**	
Medicina, n (%)	50 (76,9)
Fisioterapia, n (%)	7 (10,8)
Enfermagem ou Técnico de enfermagem, n (%)	7 (12,3)
**Titulação máxima**	
Bacharelado ou Especialização, n (%)	45 (69,3)
Mestrado ou Doutorado, n (%)	20 (30,7)

## Respostas do Questionário

Dos 65 participantes da pesquisa, 52 (80%) reportaram que permanecem em uma mesma posição (sentados ou de pé) durante a maior parte do tempo de sua jornada de trabalho em ambiente hospitalar. Em um plantão (mínimo de 12 horas) e/ou jornada típica de trabalho, 7 (10,8%) participantes permanecem em posições desconfortáveis que afetam a região lombar por 10 a 20 horas, 34 (52,3%) participantes de 5 a 10 horas, e 24 (36,9%) de 0 a 4 horas. Além disso, 47 (72,3%) relataram a necessidade de mudar de posição frequentemente durante a realização de um procedimento para ter mais conforto na região lombar.

Foi questionado se, devido a dor na região lombar, o participante evita manter-se em ortostatismo (posição de pé, ereta) ou deambular (caminhar); 9 (13,8%) responderam “Sim”, 35 (53,8%) “Não” e 21 (32,3%) “Às vezes”. Além disso, 8 (12,3%) evitam o uso de escadas ou rampas devido a dor na região lombar, enquanto 51 (78,5%) negaram evitar e 6 (9,2%) responderam “Às vezes”.

Quando questionados sobre a irradiação para membros inferiores, 4 (6,2%) responderam “Sim”, 11 (16,9%) “Às vezes” e 50 (76,9%) “Não”.

Na pergunta sobre a presença de sinais de parestesia na dor lombar, 4 (6,2%) responderam “Sim”, 56 (86,2%) “Não” e 5 (7,7%) “Às vezes”. A intensidade da dor lombar, de 0 a 10, no momento de preenchimento do questionário foi 0 (0,0%), 1 (47,7%), 2 (9,2%), 3 (15,4%), 4 (7,7%), 5 (7,7%), 6 (6,2%), 7 (4,6%), 8 (1,5%), 9 (0,0%) e 10 (0,0%). A média de intensidade de dor lombar sentida nas últimas 6 semanas pelos participantes, de 0 a 10, foi 0 (0,0%), 1 (15,4%), 2 (15,4%), 3 (15,4%), 4 (15,4%), 5 (10,8%), 6 (9,2%), 7 (7,7%), 8 (7,7%), 9 (1,5%) e 10 (1,5%).

Na questão sobre a frequência de uso de medicamentos para dor lombar nos últimos 3 meses, 45 (69,2%) responderam que não utilizam, 12 (18,5%) utilizam menos de 1 vez por semana, 4 (6,2%) de 1 a 2 vezes na semana, 1 (1,5%) de 3 a 5 vezes e 3 (4,6%) utilizam diariamente. Além disso, 32 (49,2%) participantes já consultaram com especialista devido a dor lombar e 38 (58,5%) realizam alguma intervenção complementar (fisioterapia, yoga, acupuntura, academia) devido a dor lombar ( Quadro 1 ).

## Discussão

O presente estudo proporcionou um método eficiente de avaliar a prevalência e os fatores de risco da dor lombar de caráter ocupacional em profissionais da área da saúde em nível hospitalar. Durante revisão da literatura, o questionário Rolland Morris foi considerado de boa qualidade metodológica. Assim, devido a sua maior difusão na literatura, fácil aplicabilidade e baixo custo, foi utilizado como guia para a construção do questionário sobre dor lombar ocupacional crônica entre profissionais da área da saúde que atuam em nível hospitalar, que foi utilizado para avaliar o grau de acometimento da dor lombar de origem ocupacional nesse grupo. 9 10

A dor lombar ocupacional é a principal causa de incapacidade do mundo, e uma das queixas mais prevalentes em atendimento primário no Brasil. É a complicação mais frequentemente relatada da coluna, e mais frequentemente tratada com um manejo inadequado ou ineficiente. Profissionais da área da saúde estão frequentemente expostos a fatores de risco para essa condição devido a excessiva carga de trabalho e manutenção postural inadequada. 8 Atualmente, as pesquisas sobre lombalgia ocupacional são frequentemente focadas em fatores biomecânicos e variáveis psicossociais que tiveram uma importância acrescida ao longo do tempo. 11

A dor lombar é caracterizada como uma patologia de caráter físico e psicossocial. Dessa forma, estudos de coorte prospectivos indicam que a baixa sensação de satisfação e valorização do trabalho e o excesso de tensão no trabalho associados à demanda excessiva e jornadas de longas horas mostraram um *odds ratio* (OR) significativamente aumentado para a prevalência de lombalgia. 12 Assim, os especialistas vêm orientando suas indicações de tratamento a partir de achados radiográficos (presença de artrose e/ou neuropatia) e da escala da dor, representada pelo questionário de deficiência Roland Morris. Porém, vimos que tal classificação muitas vezes não considera as peculiaridades da dor lombar de caráter ocupacional, além de ter critérios de reprodutibilidade fracos ou moderados, abrangendo uma parcela de pacientes subdiagnosticados e/ou tratados de maneira inadequada. 13 14

O diagnóstico da condição é simples, baseado num quadro clínico característico, associado a exames de imagem. Porém, a rotina de alta demanda dos profissionais da área da saúde, associada à postura corporal inadequada, tornam a dor lombar ocupacional uma condição de difícil manejo, uma vez que os fatores causais dificilmente vão se alterar. Assim, essa condição se apresenta como um desafio constante para profissionais da área da saúde no nível terciário. 15

A questão chave no manejo de qualquer doença é a definição da melhor indicação para cada tipo de tratamento. Os questionários na prática clínica têm como finalidade estratificar cada caso conforme o estágio de evolução da doença e, assim, poder guiar o melhor tratamento destes. Dessa forma, um questionário específico para a dor lombar ocupacional de profissionais da saúde em nível hospitalar é uma forma simples e barata de classificar a gravidade da condição e avaliar a presença de uma relação direta entre os fatores prejudiciais inerentes ao trabalho de longa duração em nível terciário, permanência em ortostatismo por horas prolongadas, má adesão à prática de exercícios físicos e piora do quadro descrito. Por meio destes, associados ao conhecimento do perfil sociodemográfico do grupo alvo, garante-se uma melhor intervenção preventiva e/ou terapêutica especializada. 16

Os questionários de caráter específico a doenças são geralmente considerados superiores aos seus homólogos de caráter genérico para aplicações clínicas. Por sua vez, estes são mais apropriados quando comparando diferentes condições da doença ou avaliando tipos de cuidados em todas as categorias das doenças. 17 Dessa forma, embora haja extensivas classificações e estudos sobre a prevalência da dor lombar no Brasil, percebemos que não há uma padronização exclusiva para a dor lombar de caráter ocupacional em profissionais da área da saúde em nível hospitalar.

Em estudos prévios, dores musculoesqueléticas relacionadas ao trabalho foram altamente prevalentes entre profissionais de saúde do nível hospitalar. 18 19 20 Tais quadros álgicos são frequentemente relacionados com esforço físico e estresse psicológico. Porém, em um estudo com profissionais de saúde de um hospital da Suíça, a má postura no trabalho foi o fator que mais se relacionou com as dores. 19 Da mesma forma, observou-se no presente estudo que a manutenção em uma mesma postura por um longo período de tempo e o desconforto sentido nessas posições é bastante frequente na amostra.

Apesar da notável exposição a fatores de risco para dores musculoesqueléticas, estudos apresentam uma grande variedade entre os diferentes tipos de profissionais de saúde. Outrossim, observa-se uma variabilidade de estudo para estudo de acordo com as perguntas realizadas e a métrica utilizada. 20

Este estudo possui algumas limitações. A amostra foi limitada a profissionais da área da saúde atuantes na região metropolitana de Belo Horizonte, podendo não refletir o comportamento de profissionais de outras regiões. Além disso, o questionário validado foi aplicado a 65 participantes, todavia, o objetivo primário do presente estudo foi desenvolver um questionário validado por especialistas.

## Conclusão

O uso da técnica Delphi em duas etapas contribuiu para a elaboração e validação de um questionário sobre a prevalência e fatores de risco associados a dor lombar ocupacional crônica entre profissionais de saúde que atuam em nível hospitalar. O instrumento demonstrou ser válido e de fácil aplicação. Ademais, após a aplicação da versão final do questionário, foi possível encontrar a prevalência e fatores de risco associados a lombalgia, podendo ser utilizado tanto no nível clínico quanto para fins de pesquisa.
